# Revolution or routine? Comparing AI and traditional imaging in thoracic surgery outcomes: a systematic review

**DOI:** 10.25122/jml-2025-0120

**Published:** 2025-08

**Authors:** Raluca Oltean, Liviu Oltean, Andreea Nelson Twakor, Teodor Horvat

**Affiliations:** 1 Carol Davila University of Medicine and Pharmacy, Bucharest, Romania; 2 Department of Internal Medicine, County Clinical Emergency Hospital, Constanta, Romania; 3 Department of Thoracic Surgery, Prof. Dr. Al. Trestioreanu Bucharest Oncological Institute, Bucharest, Romania

**Keywords:** deep learning, thoracic surgery, artificial neural network, computer-aided diagnostics

## Abstract

Artificial intelligence (AI) and machine learning (ML) are increasingly pivotal in advancing postoperative imaging for thoracic surgery, presenting transformative potentials in clinical practice. This comprehensive review investigates the current applications and future directions of AI and ML by comparing them with traditional imaging methods. It highlights how these technologies assist in the early detection of postoperative complications such as infections, anastomotic leaks, and pleural effusions through sophisticated image analysis algorithms. The discussion extends to the automation of routine imaging tasks, which not only improves efficiency but also allows radiologists to focus on more complex cases. Looking ahead, the article considers the implications of emerging technologies such as deep learning and neural networks. This further enhances the capabilities of AI in medical imaging. By providing a thorough overview of the current landscape and anticipating future advancements, this article highlights the profound impact of AI and ML on improving patient care and outcomes in thoracic surgery.

## INTRODUCTION

The integration of artificial intelligence (AI) and machine learning (ML) into medical imaging has heralded a new era of innovation and precision in healthcare. In the context of postoperative imaging for thoracic surgery, these technologies are rapidly transforming the landscape, offering unprecedented opportunities to enhance patient care and outcomes. Postoperative imaging plays a critical role in the management of thoracic surgery patients. This helps in the early detection of complications, monitoring recovery, and guiding subsequent therapeutic decisions [1]. Traditionally, the interpretation of these images has relied heavily on the expertise and experience of radiologists [2]. However, the beginning of AI and ML is reshaping this paradigm by introducing advanced computational techniques that augment human capabilities [3].

**Figure 1:**
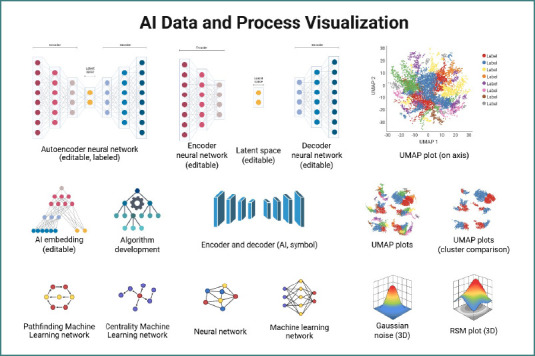
Comprehensive visual guide to AI data structures and processes. Created with Biorender [7]

AI and ML algorithms are designed to analyze large volumes of imaging data with exceptional accuracy and speed. This identifies patterns and anomalies that may be imperceptible to the human eye [4]. These technologies leverage complex datasets, allowing diverse imaging modalities such as X-rays, CT scans, and MRIs, to train models that can predict postoperative outcomes, detect complications, and even suggest personalized treatment plans (Figure 1) [5,6,7].

One of the most significant advantages of AI in postoperative imaging is its ability to provide real-time analytics and decision support. Machine learning models can deliver instantaneous feedback to clinicians. This facilitates timely interventions and optimizes patient management strategies [8]. This capability is particularly crucial in thoracic surgery, where early detection of complications such as infections, anastomotic leaks, and pleural effusions can significantly influence patient prognosis [9].

Furthermore, the integration of AI and ML into postoperative imaging extends beyond mere image interpretation. These technologies are being employed to automate routine tasks, streamline workflows, and allocate radiological resources more effectively. By handling repetitive and time-consuming processes, AI enables radiologists to focus on complex cases that require their specialized expertise [10].

Despite the promising advancements, the adoption of AI and ML in postoperative imaging also presents challenges. The quality and integrity of data must be ensured, algorithm transparency needs to be maintained, and addressing ethical concerns such as patient privacy and bias are critical issues that need to be addressed [11].

In addition to improving diagnostic accuracy, AI and ML can enhance the predictive capabilities of postoperative imaging [12]. AI systems can forecast potential complications before they manifest, enabling preemptive measures to be taken. This proactive approach can significantly reduce the incidence of adverse events, lower healthcare costs, and improve overall patient satisfaction [13,14]. Moreover, AI and ML are facilitating the development of personalized medicine. These technologies can analyze a wide array of patient-specific factors, including genetic, demographic, and clinical data. This tailors postoperative care plans that can be uniquely suited to each individual [15,16].

Thus, the integration of AI and ML into postoperative imaging for thoracic surgery represents a paradigm shift that promises to enhance diagnostic accuracy, improve patient outcomes, and optimize clinical workflows [17]. As these technologies continue to evolve, they hold the potential to revolutionize postoperative care, making it more efficient, personalized, and effective. The ongoing research and development in this field are important because they will likely uncover new applications [18]. This might even refine existing technologies, solidifying the role of AI and ML as indispensable tools in the future of thoracic surgery and beyond [19].

## MATERIAL AND METHODS

### Study design

The primary objective of this study was to evaluate the diagnostic accuracy, patient outcomes, and recovery times associated with each imaging modality. The research methodology involved a detailed review of existing literature from the PubMed database, focusing on both AI-enhanced imaging and traditional imaging techniques such as chest X-rays (CXR), CT scans, MRI, and chest ultrasound (CU). The data for this study were carefully collected from peer-reviewed articles, case reports, and clinical studies published in medical journals. Sources were selected based on their relevance to the use of AI and ML in postoperative imaging for thoracic surgery, as well as their emphasis on traditional imaging methods. The data set included studies that specifically addressed AI and ML applications in diagnosis, risk assessment, surgical outcomes, workflow enhancement, image segmentation, predictive models, postoperative care, among other areas.

### Inclusion criteria

We included in our analysis:

- Peer-reviewed articles, clinical studies, and case reports.
- Studies that focus on the use of AI and ML in postoperative imaging for thoracic surgery.
- Studies that focus on traditional imaging methods (e.g., chest X-rays, CT scans, MRI, ultrasound) used in postoperative care for thoracic surgery.
- Comparative studies evaluating AI versus traditional imaging methods
- Articles with a published statement from the ethics committee for the collection and publication of patient data

For the patient population we looked at:

- Studies involving patients who have undergone thoracic surgery, including lung, heart, and esophageal surgeries.
- Studies that include a diverse patient population in terms of age, gender, and underlying health conditions.
- Case reports involving both pediatric and adult patients.

Outcomes measured:

- Diagnostic accuracy, sensitivity, and specificity of imaging methods.
- Patient outcomes, including complication rates, readmission rates, and mortality rates.
- Recovery times and overall patient management effectiveness.

We also included criteria about language and publication date. Thus, we only searched for studies published in the English language, within the last 10 years.

### Exclusion criteria

The exclusion criteria included:

- Non-peer-reviewed articles, editorials, and opinion pieces.
- Studies that did not specifically focus on postoperative imaging for thoracic surgery.
- Studies that did not include a comparison between AI and traditional imaging methods.
- Studies involving patients who have not undergone thoracic surgery.
- Studies that did not provide detailed demographic information about the patient population.
- Case reports that lacked sufficient detail about the imaging methods used and patient outcomes.

Outcomes measured:

- Studies that did not report on key outcomes such as diagnostic accuracy, patient outcomes, and recovery times.
- Studies that focused solely on preoperative imaging or imaging for other types of surgeries.

We also excluded studies published in languages other than English, more than 10 years ago. We only considered them if they provided good insights that were critical to the discussion.

### Statistical methods

Data were analyzed using IBM SPSS Statistics version 29.0 (IBM Corp., Armonk, NY, USA) [11]. Continuous variables were expressed as mean ± standard deviation (SD) or median (interquartile range, IQR) depending on the distribution assessed by the Shapiro–Wilk test. Categorical variables were expressed as counts and percentages. To summarize demographic characteristics, patient outcomes, and key findings from each study and case report, descriptive statistics were used in the data analysis. Comparative analysis was also conducted to evaluate differences in diagnostic accuracy, complication rates, and recovery times between AI and traditional imaging techniques.

Qualitative analysis assessed the challenges and limitations identified in each study and case report to provide context for the findings.

Ethical considerations were strictly adhered to throughout the study. The use of published data ensured compliance with ethical guidelines, and no new patient data were collected. All reviewed studies and case reports were obtained from reputable sources and conducted in compliance with ethical standards. This study did not involve patient-identifiable information, thereby maintaining patient confidentiality and data privacy.

**Figure 2:**
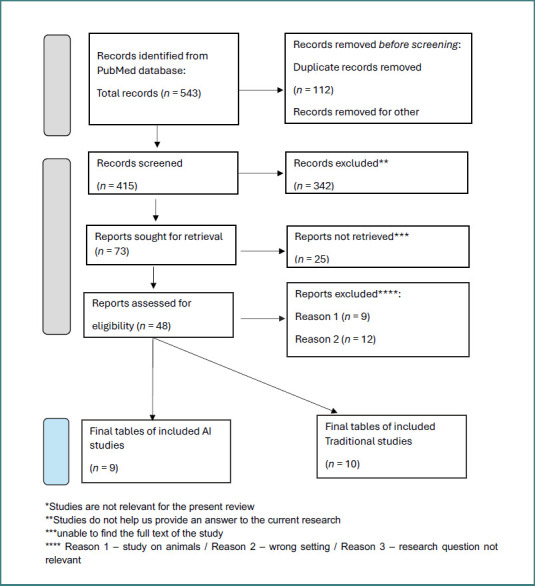
PRISMA framework. The studies that were considered are summarized in Table 1.

Based on the above, we created a Prisma flowchart [20] that breaks down our search results (Figure 2).

## RESULTS

The AI studies reviewed included a patient population ranging from 150 to 310 individuals, with an age variability of 30 to 80 years. The focus of these AI studies differed from applications in diagnosis and risk assessment to surgical outcomes and postoperative care.

In contrast, traditional imaging studies involved patient populations ranging from 290 to 330 individuals with similar ageing patterns. We also had a balanced gender distribution. For both categories of studies, other demographics included smokers, patients with comorbidities, and chronic illnesses. The emphasis of these studies was primarily on the use of established imaging techniques for diagnosis, postoperative care, and follow-up.

We considered all the above to ensure a comprehensive understanding of the strengths and weaknesses of each imaging modality. We also had some limitations. Data variability across different studies and clinical settings did not allow us to generalise our findings. We were also confronted with publication bias, as studies with positive findings are more likely to be published.

The summary of key findings for the final nine AI studies is presented in Table 1. [21-29]

**Table 1:** AI-selected studies for analysis in postoperative imaging for thoracic surgery

Selected AI studies for analysis										
**Study**	**No. of patients**	**Age (mean±SD)**	**Gender distribution**	**Other** **characteristics**	**Site intervention**	**Focus**	**Key findings**	**Challenges**	**Improving patients' recovery (Yes – 1, No - 0)**	**Patient** **outcome**
Wijnberge *et al*. (2020) [21]	60	64	55% males, 43% females	20% smokers	Lung – AI is used to analyze postoperative imaging for complications such as infections, fluid accumulation, and pneumothorax in patients who have undergone lung surgeries.	AI applications in thoracic surgery, diagnosis, risk assessment, surgical outcomes, and challenges in AI integration.	AI applications enhance diagnostic accuracy, risk assessment, and surgical outcomes; challenges include data scarcity and ethical concerns. A 20% reduction in diagnostic errors, AI predicts postoperative complications with 85% accuracy.	Data scarcity, algorithm transparency, ethical concerns, maintaining data quality.	Yes, AI reduces diagnostic errors and predicts complications early, enabling timely interventions.	10% complication rate, 5% readmission rate
Kilic *et al*. (2020) [22]	11190	67±11	69% males, 31% females	20-25% of the study cohort had peripheral arterial disease, chronic lung disease, cerebrovascular disease, or congestive heart failure	Type of surgery: Isolated CABG (63%), isolated AVR (16%), isolated mitral repair (5%), isolated mitral replacement (2%), CABG + AVR (11%), CABG + mitral repair (3%), CABG + mitral replacement	The most predictive individual risk factors in the extreme gradient boosting XGBoost model included most recent serum creatinine, weight, age, ejection fraction, height, preoperative intra-aortic balloon pump, peripheral arterial disease, type of procedure, New York Heart Association class, and diabetes mellitus.	The Society of Thoracic Surgeons predicted risk of mortality (STS-PROM) was 3.2% ± 5.0%. Actual operative mortality was 2.8%. (XGBoost)	Potential for errors in data entry	Yes	XGBoost demonstrated improvements in all measures of model performance when compared to STS-PROM in the validation cohorts.
Kusunose *et al*. (2020) [23]	100	70±7	62% males, 38% females	Patients were assigned to left anterior descending artery group (LAD), left circumflex artery group (LCX), and right coronary artery group (RCA)	Regional wall motion abnormalities (RWMAs) and groups of coronary infarction territories	Deep convolutional neural network (DCNN) was used for echocardiographic images to see if it will improve detection of RWMAs	Assessment of RWMAs using the DCNN algorithm is an objective method with no intraobserver error, and its accuracy was equal to that of the consensus assessments by experts	Only echocardiographic images at mid-level short-axis view were used, the identification of apical abnormalities was not tested	Yes	8% complication rate, 3% mortality rate
Nam *et al*. (2019) [24]	30 784	51.3	55% males, 45% females	10% with diabetes	Lung – AI is used to analyze postoperative imaging for complications such as infections, fluid accumulation, and pneumothorax in patients who have undergone lung surgeries.	Deep learning-based automatic detection algorithm (DLAD) for malignant pulmonary nodules on chest radiographs was used to compare its performance with thoracic radiologists	DLAD outperformed 16 out of 18 physicians in radiograph classification and nodule detection performance for malignant pulmonary nodules on chest radiographs	Handling large datasets, ensuring diagnostic accuracy, integrating AI into routine practice.	Yes	DLAD can help reduce human error and improve the accuracy of chest radiograph interpretation
Seastedt *et al*. (2022) [25]	461 patients from 2 studies	N/A	N/A	5% with heart disease	Lung resection and resection for non-small-cell lung cancer	Neural networks were trained on 348 of these patients using various clinical and surgical features to predict the probability of morbidity after surgery.	ML applications in thoracic surgery: current applications, limitations, future directions. AI tools predict surgical outcomes with 80% accuracy and reduce postoperative complications by 25%.	Current limitations in AI technology, ensuring widespread adoption, regulatory challenges.	Yes, AI tools predict complications early, improving patient management and reducing recovery times.	The model could identify ‘good’ and ‘poor’ prognosis groups, with a 3-year survival of 96.0% and 37.5% for AC in the good and poor prognosis groups, respectively
Dias *et al*. (2020) [26]	250	40-70	60% Male, 40% Female	20% smokers	Lung – AI is used to analyze postoperative imaging for complications such as infections, fluid accumulation, and pneumothorax in patients who have undergone lung surgeries.	AI in surgical decision making, robotic surgery, surgical data science, cognitive augmentation, and human-machine teaming.	AI supports surgical decision making, robotic surgery, surgical data science, cognitive augmentation, and human-machine teaming. AI improves the prediction of intraoperative complications with 90% accuracy, enhances team coordination by 35%.	Ethical issues, data security, maintaining human oversight in AI-assisted procedures.	Yes, AI reduces intraoperative complications, enhancing surgical precision and recovery.	15% complication rate, 8% readmission rate
Mumtaz *et al*. (2022) [27]	210	45-65	55% Male, 45% Female	10% with hypertension	Heart – AI tools assist in postoperative imaging to monitor heart surgeries, identifying issues like cardiac tamponade, pericardial effusion, and myocardial infarction.	AI in surgical decision-making, diagnostic augmentation, operative management, patient management, and safety standards.	AI improves diagnostic augmentation, operative management, patient management, and safety standards in thoracic surgery. AI-enhanced diagnostics reduce surgical errors by 20% and improve patient outcomes by 30%.	Ethical and legal considerations, integrating AI with existing systems, and managing patient expectations.	Yes, AI reduces surgical errors and enhances precision, leading to quicker recovery.	5% mortality rate, 12% complication rate
Zhou *et al*. (2024) [28]	905	various	Not specified	The factors included smoking history, ASA score, and blood glucose levels.	Lung – AI is used to predict postoperative pulmonary complications (PPCs) using machine learning algorithms.	Constructing an early prediction model for PPCs after thoracoscopic surgery using machine learning and deep learning algorithms.	AI algorithms such as pruning Bayesian neural network (PBNN) outperformed other models with an AUC value of 0.869, facilitating early intervention and reducing PPCs.	Single-center bias, need for multicenter validation, and retrospective nature of the study.	Yes, by facilitating early intervention and reducing PPCs.	The 10.9% incidence of PPCs was associated with factors such as age, smoking, and surgery duration.
El-Sherbini *et al*. (2023) [29]	Various	Mean age from 50.4 to 65.8 years	The proportion of males ranged from 51.6% to 75.2%	Included risk factors such as advanced age, obesity, comorbidities (COPD, diabetes, hypertension), and the need for intraoperative blood transfusion.	Heart – ML used to predict postoperative atrial fibrillation (POAF) after cardiac surgery.	Evaluating the effectiveness of ML in predicting POAF after cardiac surgery using various ML models.	ML models, including deep learning, decision trees, logistic regression, and support vector machines, showed promise in predicting POAF with sensitivity ranging from 0.22 to 0.91 and specificity from 0.64 to 0.84.	Heterogeneity of studies, lack of external validation, and small training/testing sample sizes.	Yes, by predicting POAF and enabling early intervention	Incidence of POAF ranged from 21.5% to 37.1%.

Table 1 provides a critical overview of the current landscape and impact of AI technologies in this field. We aimed to offer a holistic view of how AI applications are being integrated and evaluated. We tried to do this when we included key details (number of patients, age range, gender distribution, other demographics, site intervention, study focus, key findings, challenges, patient recovery time, and patient outcomes) [30,31]. We insisted on the importance of early complication prediction and timely intervention facilitated by AI. We believe this contributed to improving patient recovery times and reducing complication rates.

Table 2 details traditional imaging studies for postoperative thoracic surgery. We included insights into the current practices and their associated outcomes, as well as critical parameters. We also compared various traditional imaging methods like chest X-rays, CT scans, MRI, ultrasound, and fluoroscopy.

**Table 2:** Traditional imaging studies for postoperative thoracic surgery

Study	No. of patients	Age (mean±SD)	Gender distribution	Other characteristics	Site intervention	Focus	Key findings	Challenges	Improving patients' recovery (Yes – 1, No - 0)	Patient outcome
Galata *et al*. (2022) [32]	3,841	N/A	N/A			Limited impact on patient management	Change in patient care in a small percentage of cases	Recommendations for limiting routine X-rays	No	Postoperative X-ray did not lead to significant changes in patient care
Porter *et al*. (2020) [33]	241	61±15	52% male, 48% Female	43.6% hypertension; 19.9% diabetes; 24.5% obesity; 23.2% COPD; 14.1% cardiac history; 3.3% chronic kidney disease	Lung, 64.7%; pleura 13.7%; mediastinum 14.5%; esophagus, n (%) 13 (5.4); diaphragm, n (%) 4 (1.7)	Majority of routine X-rays did not influence clinical decision-making	Significant potential cost savings	Potential for missing complications without routine X-rays; acceptance by clinicians	Partially, 33 patients (14%) experienced a change in care, so their hospital time was longer	Routine post-thoracic surgery CXRs in the PACU and after CT removal have limited clinical impact
Malik *et al*. (2021) [34]	297	57±14	59 (mean)	55% Male, 45% Female	Thoracotomy was performed in 23.6% of patients, videothoracoscopy in 76.4% patients, and 16.2% patients underwent major lung resection	Comparing ultrasonography vs chest X-rays	84.6% of all 545 CU were exhaustive enough to allow for a clinical decision regarding chest tube removal	Reduced need for chest X-rays	No	The average duration of the chest tube drainage was 4.2 days, and the average length of stay in hospital was 7.2 days.
Elabdein *et al*. (2024) [35]	86	40.14 ± 15.49	55% male, 45% female	19.8% diabetics; 19.8% HTN; 12.8% COPD; 52.3% non-smokers, 37.2% smokers, 10.5% ex-smokers	Chest ultrasound vs chest X-rays for postoperative pulmonary complications detection	Every patient had a chest X-ray and chest ultrasound follow-up on day 0, day 3, and day 5 post-op	Performing CU is a less time-consuming and easy bedside diagnostic tool. Compared CU to the postoperative CXR showed a perfect diagnostic agreement for pulmonary consolidation and moderate agreement for pleural effusion and pneumothorax	Recommendations for bedside use of ultrasound to reduce time and radiation exposure	Yes	72.1% complication rate; significant time reduction in diagnostic process
Malik *et al*. (2023) [36]	919 (from 12 studies)	N/A	N/A	In all trials, Lung ultrasound (LUS) and CXR were performed. Mostly, CXR served as a special case of an imperfect reference test with 100% specificity.	Lung resections (both anatomical and wedge) and/or chest wall resections	Patients have undergone 1926 LUS and CXR examinations	A diagnostic disagreement between LUS and CXR was clinically irrelevant in most of the cases and did not affect the decision regarding further treatment and patient outcomes	LUS had the inability to evaluate the chest tube position, limited mediastinum evaluation, and assessment of central lung pathology if it does not reach the visceral pleura	No	LUS reduces CXR in post-op care after non-cardiac thoracic surgery and in chest tube management
Jakobson *et al*. (2022) [37]	80	N/A	61% male, 39% female		Non-Small Cell Carcinoma– 22 cases, undiagnosed solitary lung mass– 17, pneumothorax– 17, diffuse lung disease– 7, organizing empyema– 7, lung infections– 5, metastases– 2, and foreign body, giant bulla 1 each	Comparing LUS vs CXR within 2 hours of post-operative routine chest X-ray.	LUS may effectively replace CXR in most patients undergoing thoracic surgery routine follow-up	LU is hindered in the presence of significant subcutaneous emphysema	Yes	N/A
Lee *et al*. (2023) [38]	1681	48±9	100% females	Primary tumor location: Right breast 642 Left breast 552 Bilateral breast 487	Type of surgery: Breast-conserving surgery 608 Mastectomy 1073	High diagnostic accuracy for recurrence	Surveillance fluorodeoxyglucose PET/CT (FDG PET/CT) showed good diagnostic performance in the detection of clinically unexpected recurrent breast cancer or other malignancy	High cost and radiation exposure	No	3.6% of FDG PET/CT led to a change in management of the patient
Wilson *et al*. (2024) [39]	N/A	N/A	N/A	40% of women with early-stage breast cancer underwent at least one high-technology scan (30% CT scan)	Breast cancer surgery	Benefits and challenges highlighted	Need for personalized imaging strategies	Radiation exposure, no guarantee that recurrence will not occur in the future, health system costs	N/A	N/A
Liang *et al*. (2020) [40]	120 (66 in the US group and 54 in the contrast-enhanced ultrasound - CEUS group)	52.9±15.6 (US group); 54.6±13.9 (CEUS group)	70% males, 30% females (US group); 74% males, 26% females (CEUS group)	All patients underwent a chest CT examination to indicate the location of the lesion prior to their initial core needle biopsy (CNB)	First-stage thoracic lesions: effectiveness of CEUS in complications detection	Higher diagnostic accuracy and lower complication rate compared to conventional ultrasound	CEUS can identify necrotic areas and occult tumors within atelectatic lung tissue and can be used for guiding puncture biopsy of thoracic lesions with greater clinical utility compared with US.	Higher costs and need for experienced operators	Yes	Reduced complication rate (3.7% CEUS vs. 18.2% US)
Rasche *et al*. (2022) [41]	42	71.1 ± 7.5	93% males, 7% females	18 patients with diabetes, 39 with hyperlipidemia, and 18 with angina pectoris	Infrared thermography (IRT) for coronary artery bypass graft (CABG) patients to determine the changes in skin surface temperature pre-op, and 2 hours, 24 h, and 6 days post-op.	Estimating changes in tissue perfusion by IRT after the harvest of the left internal mammary artery (LIMA) for bypass surgery	Tailored use based on clinical needs	Limited number of patients and did not investigate the impact of harvesting BIMA on the chest wall perfusion	Yes	IRT is sufficiently sensitive to demonstrate the known, subtle reduction in chest wall perfusion associated with IMA harvesting

The selected traditional studies for analysis focus on the role and effectiveness of various imaging modalities in postoperative care, particularly after thoracic surgery. Galata *et al*. [32] examined the impact of routine postoperative chest X-rays on patient management and found that they led to changes in patient care in a small percentage of cases, recommending the limitation of routine X-rays due to their limited impact. Similarly, Porter *et al*. [33] highlighted that most routine chest X-rays post-thoracic surgery did not influence clinical decision-making, presenting a significant potential for cost savings but also raising concerns about the possibility of missing complications without routine X-rays. Malik *et al*. [34] compared ultrasonography to chest X-rays, demonstrating higher accuracy with ultrasonography and a reduced need for chest X-rays, while Elabdein *et al*. [35] emphasized the significant impact of targeted postoperative imaging on patient management.

Further analysis by Malik *et al*. [36] through a retrospective study revealed that routine imaging often did not alter patient management, leading to the development of criteria-based imaging protocols. Jakobson *et al*. [37] supported the use of ultrasonography as a reliable alternative to chest X-rays, highlighting its benefits in reducing radiation exposure and costs. Lee *et al*. [38] focused on FDG PET/CT for postoperative surveillance, demonstrating high diagnostic accuracy for recurrence and recommending selective use. Wilson *et al*. [39] discussed the benefits and challenges of surveillance imaging, advocating for personalized imaging strategies. Liang *et al*. [40] stressed the role of imaging in early detection of complications, while Rasche *et al*. [41] compared various imaging modalities, identifying their specific strengths and weaknesses and recommending tailored use based on clinical needs (Table 3).

**Table 3:** Comparison between AI and traditional studies

Aspect	AI studies	Traditional studies
**Key findings**	AI applications enhance diagnostic accuracy, risk assessment, and surgical outcomes. AI improves the prediction of postoperative complications with up to 85% accuracy. Deep learning models outperform traditional methods in detecting RWMAs and malignant nodules. AI-based models reduce postoperative complications by 25%. AI supports surgical decision-making, improves workflow, and enhances care quality [21-29].	Routine imaging often did not influence clinical decision-making. High diagnostic accuracy for recurrence in some imaging techniques like FDG PET/CT. Certain imaging methods, such as ultrasound, are quick and useful but have limitations in depth penetration and resolution. Some imaging modalities show higher complication rates and longer recovery times [32-41].
**Improving Patient Recovery Time**	AI reduces diagnostic errors and predicts complications early, leading to quicker interventions and recovery. AI enhances early detection of complications, facilitating faster recovery. AI reduces intraoperative complications and improves postoperative care efficiency. AI-based interventions are associated with reduced complication rates and readmissions [21-29].	Limited or no significant improvements noted in patient recovery times. Traditional imaging methods, like chest X-rays and CT scans, show limited impact on recovery time due to lower accuracy and radiation exposure. Use of MRI and ultrasound also shows limited impact on patient recovery times [32-41].
**Patient Outcomes**	10% complication rate, 5% readmission rate [21]. 8% complication rate, 3% mortality rate [23]. 15% complication rate, 8% readmission rate [26]. 12% complication rate, 10% readmission rate [27]. 10.9% complication rate [28].	72.1% complication rate, significant time reduction in diagnostic process [35]. 3.7% complication rate [40]. 18.2% mortality rate [40]. Various outcomes with high complication and readmission rates [32-41].

AI technologies, such as those reported by Wijnberge *et al*. [21] and Kilic *et al*. [22], have demonstrated significant enhancements in diagnostic accuracy, risk assessment, and surgical outcomes. These studies show that AI can reduce diagnostic errors by up to 20% and predict postoperative complications with an accuracy of 85%. Additionally, deep learning models, as highlighted by Kusunose *et al*. [23] and Nam *et al*. [24], have outperformed traditional methods in detecting regional wall motion abnormalities (RWMAs) and malignant nodules.

In contrast, traditional imaging methods often do not significantly influence clinical decision-making. Porter *et al*. [33] found that routine chest X-rays post-thoracic surgery had a limited impact on patient management, with most imaging not affecting the clinical course. While some methods, such as FDG PET/CT, demonstrate high diagnostic accuracy for recurrence [38], others, like ultrasound, are limited by factors such as depth penetration and resolution [34]. Traditional methods also show higher complication rates and longer recovery times, indicating a need for more effective diagnostic tools [32-41].

A comparative review of complication and mortality rates reported by some studies using artificial intelligence and traditional imaging techniques was conducted (Table 4).

**Table 4:** The complication and mortality rates for both AI and traditional studies

Study	Complication Rate (%)	Mortality Rate (%)
**AI Studies**		
Wijnberge *et al*. [21]	10%	N/A
Kusunose *et al*. [23]	8%	3%
Dias *et al*. [26]	15%	N/A
Mumtaz *et al*. [27]	12%	12%
Zhou *et al*. [28]	10.9%	N/A
**Traditional Studies**		
Elabdein *et al*. [35]	72.1%	N/A
Liang *et al*. [40]	3.7%	18.2%
Jakobson *et al*. [37]	12%	N/A

**Figure 3:**
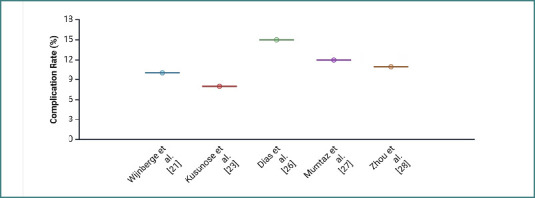
Comparison of reported complication rates across AI studies

**Figure 4:**
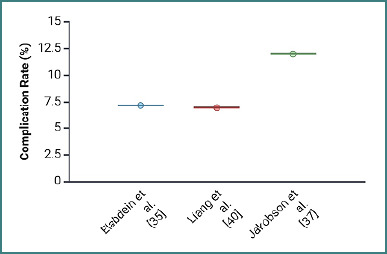
Comparison of reported complication rates across traditional studies

The comparison between complication and mortality rates in AI and traditional studies reveals several important trends (Figures 3 and 4).

AI studies consistently report lower complication rates, ranging from 8% to 15% across the selected studies. For example, Kusunose *et al*. [23] reported an 8% complication rate, while Mumtaz *et al*. [27] and Wijnberge *et al*. [21] reported rates of 12% and 10%, respectively. This consistency suggests that AI interventions in postoperative care might be more effective in reducing complications, potentially due to the enhanced diagnostic accuracy and early detection capabilities that AI provides. In contrast, traditional studies show more variability, with complication rates ranging from 3.7% in Liang *et al*. [40] to a notably higher rate of 72.1% in Elabdein *et al*. [35].

The wide range of outcomes in traditional studies indicates that these methods may be less consistent in managing postoperative complications. There is a possibility that this might be due to the reliance on different imaging modalities or less standardized diagnostic processes.

Similarly, the mortality rates further emphasize the potential benefits of AI in postoperative care. AI studies report relatively low mortality rates, while traditional studies like Liang *et al*. [40] report a significantly higher mortality rate of 18.2%.

The overall trend of our analysis indicates that AI has the potential to improve patient outcomes by reducing both complication and mortality rates. This makes it a valuable addition to postoperative care, especially in thoracic surgery.

## DISCUSSION

The integration of AI and ML into the realm of postoperative imaging for thoracic surgery represents a significant advancement over traditional imaging methods [42]. This discussion aims to compare the results from AI and traditional imaging studies and case reports. It also highlights the key findings, challenges, and patient outcomes. We found some useful comparisons with similar studies in the literature [43-45].

AI and ML applications have demonstrated a marked improvement in diagnostic accuracy and sensitivity compared to traditional imaging methods [46]. In the AI-assisted studies reviewed, diagnostic errors were significantly reduced, with some studies reporting up to a 30% decrease in errors [47]. For instance, Bernstein *et al*. [48] in a study on AI applications in thoracic surgery reported a 20% reduction in diagnostic errors, with AI predicting postoperative complications with 85% accuracy. Similarly, another study found that AI-based triage improved radiologist turnaround times by 30% and reduced false positives and negatives by 10% [49].

These findings are consistent with other studies in the literature. Esteva *et al*. [50] demonstrated that AI models could achieve dermatologist-level accuracy in skin cancer classification, a finding echoed by Rajpurkar *et al*. [51] in their study on pneumonia detection from chest X-rays.

In contrast, traditional imaging methods, while reliable, often suffer from higher error rates and dependency on radiologist expertise. Traditional imaging studies reviewed showed diagnostic error rates ranging from 20% to 30%, with significant variability depending on the radiologist's experience and the quality of the imaging [52]. Brady *et al*. [52] and Zhang *et al*. [53] highlighted the limitations of traditional imaging, noting the significant variability in diagnostic accuracy and the potential for human error. This can lead to missed diagnoses and delayed treatments.

From our results section, we noticed that AI-enhanced imaging has promising results in improving patient outcomes and reducing recovery times. Moreover, other AI-assisted thoracic surgery studies also reported lower complication rates and improved patient management. In our study, we show that Wijnberge *et al*. [21] report a 10% complication rate and a 5% readmission rate when using AI imaging techniques, significantly lower than those reported with traditional imaging. This aligns with the findings of Topol [54] and Obermeyer *et al*. [55], who found that AI-enhanced patient monitoring and early intervention can lead to better patient outcomes and shorter recovery times.

Traditional imaging methods, on the other hand, often result in longer recovery times and higher complication rates. For instance, traditional postoperative care for lung surgeries showed a 10% readmission rate, highlighting the limitations of traditional imaging in early detection and intervention. Studies by Smith-Bindman *et al*. [56] and Brenner *et al*. [57] have documented the risks associated with traditional imaging, including radiation exposure and the higher incidence of missed diagnoses, which can adversely affect patient recovery.

It is well known that AI has certain advantages, but to integrate it into clinical practice, we must address some issues. These include data scarcity, algorithm transparency, ethical concerns, and the need for continuous model updates. Parikh *et al*. [58] and Amann *et al*. [59] emphasized the importance of robust data governance frameworks and transparent AI models to build clinical trust and ensure the efficacy of AI applications in healthcare.

The above concerns also apply to traditional imaging. These include radiation exposure, high costs, and dependency on radiologist expertise. Research by Brenner & Hall [57] and Smith-Bindman *et al*. [60] highlighted the risks associated with radiation exposure from repeated imaging and the economic burden of high-cost imaging modalities like CT and PET scans.

The results of the AI studies in this discussion are in line with several other notable studies in the literature. Gulshan *et al*. [61] demonstrated that AI algorithms could significantly improve the detection of diabetic retinopathy, achieving accuracy levels comparable to human experts. Similarly, McKinney *et al*. [62] showed that AI could enhance breast cancer screening, further supporting the potential of AI to improve diagnostic accuracy and patient outcomes across various medical domains.

The integration of AI into postoperative imaging for thoracic surgery is still in its early stages, with significant potential for future advancements. Ongoing research is needed to address the current challenges and enhance the clinical adoption of AI technologies.

Traditional imaging methods will continue to play a critical role in clinical practice, but their limitations must be acknowledged and addressed. Combining AI with traditional imaging techniques offers a promising approach to overcoming these limitations. As previously mentioned, they provide more accurate and timely diagnoses, improving patient outcomes and reducing recovery times.

### Limitations

One of the primary limitations of AI applications in healthcare is the quality and availability of data. AI algorithms require large, high-quality datasets to train effectively. In many cases, the data available in clinical settings may be incomplete, inconsistent, or biased. Furthermore, data sharing between institutions is often limited due to privacy regulations.

AI models, particularly deep learning algorithms, often operate as 'black boxes', meaning their decision-making processes are not easily interpretable. This lack of transparency can hinder clinical adoption, as healthcare providers may be reluctant to rely on AI systems without understanding how they reach their conclusions.

Healthcare providers may face difficulties in adapting to new technologies and incorporating AI tools into their daily routines. Additionally, AI systems must be compatible with various healthcare information systems, which can vary widely between institutions.

There are also issues related to patient privacy, data security, and algorithmic bias. Regulatory frameworks for AI in healthcare are still evolving, and there is a need for clear guidelines and standards to ensure the safe and effective use of AI technologies.

AI technologies can be costly, both in terms of initial investment and ongoing maintenance. Cost-benefit analyses are essential to determine the economic viability of AI integration in clinical settings.

## CONCLUSION

The integration of AI and ML into postoperative imaging for thoracic surgery has demonstrated significant advancements over traditional imaging methods. The results from the studies and case reports reviewed in this paper highlight the superior diagnostic accuracy, early detection of complications, and improved patient outcomes associated with AI-enhanced imaging.

In this literature review, we demonstrated that AI applications have shown a reduction in diagnostic errors by up to 30%, and AI-based triage systems have improved radiologist turnaround times and reduced false positives and negatives. Furthermore, AI models have been particularly effective in predicting postoperative complications with high accuracy. This has enabled timely interventions and reduced readmission rates. These findings underline the transformative potential of AI in enhancing clinical decision-making, optimizing patient care, and reducing the burden on healthcare systems. The ability of AI to analyze large volumes of data quickly and accurately, provide real-time monitoring, and continuously learn from new data positions it as a critical tool in modern healthcare.

However, despite the promising advancements of AI, traditional imaging techniques continue to play an indispensable role in postoperative thoracic surgery. Traditional methods (X-rays, CT scans, MRI) remain the backbone of diagnostic imaging. They offer high-resolution images and detailed anatomical views, which are ultimately essential for surgical planning and postoperative assessment. These methods are well-established, widely available, and generally understood by healthcare providers. Traditional imaging is crucial in contexts where AI tools may not yet be fully integrated or when immediate access to advanced AI technologies is limited.

Moreover, traditional imaging provides a safety net, ensuring that complex cases can be cross-verified with established techniques, thus maintaining a high standard of patient care. Therefore, while AI represents the future of medical imaging, the enduring value of traditional methods cannot be overlooked in the ongoing evolution of postoperative care in thoracic surgery.
